# Chaperone mediated autophagy contributes to the newly synthesized histones H3 and H4 quality control

**DOI:** 10.1093/nar/gkab1296

**Published:** 2022-01-17

**Authors:** Juan Hormazabal, Francisco Saavedra, Claudia Espinoza-Arratia, Nicolas W Martinez, Tatiana Cruces, Iván E Alfaro, Alejandra Loyola

**Affiliations:** Centro Ciencia & Vida, Fundación Ciencia & Vida, Santiago, Chile; Centro Ciencia & Vida, Fundación Ciencia & Vida, Santiago, Chile; Facultad de Medicina y Ciencia, Universidad San Sebastián, Santiago, Chile; Centro Ciencia & Vida, Fundación Ciencia & Vida, Santiago, Chile; Centro Ciencia & Vida, Fundación Ciencia & Vida, Santiago, Chile; Centro Ciencia & Vida, Fundación Ciencia & Vida, Santiago, Chile; Centro Ciencia & Vida, Fundación Ciencia & Vida, Santiago, Chile; Instituto de Ciencias e Innovación en Medicina, Facultad de Medicina Clínica Alemana Universidad del Desarrollo, Santiago, Chile; Centro Ciencia & Vida, Fundación Ciencia & Vida, Santiago, Chile; Facultad de Medicina y Ciencia, Universidad San Sebastián, Santiago, Chile

## Abstract

Although there are several pathways to ensure that proteins are folded properly in the cell, little is known about the molecular mechanisms regulating histone folding and proteostasis. In this work, we identified that chaperone-mediated autophagy (CMA) is the main pathway involved in the degradation of newly synthesized histones H3 and H4. This degradation is finely regulated by the interplay between HSC70 and tNASP, two histone interacting proteins. tNASP stabilizes histone H3 levels by blocking the direct transport of histone H3 into lysosomes. We further demonstrate that CMA degrades unfolded histone H3. Thus, we reveal that CMA is the main degradation pathway involved in the quality control of histone biogenesis, evidencing an additional mechanism in the intricate network of histone cellular proteostasis.

## INTRODUCTION

Histone proteins play pivotal roles in eukaryotic cells, compacting the DNA into chromatin and regulating gene expression by changing the DNA accessibility. As a cell divides, DNA and chromatin are duplicated and assembled in such a way that the newly replicated DNA is accompanied by the recycled parental histones and by the synthesis of new histones which reaches a peak in S-phase (1,2). Upon histone synthesis, H3 and H4 undergo a maturation cascade that prompts correct folding and establishes post-translational modifications (1,3–10). Throughout this maturation pathway, histones interact with different chaperones and histone binding proteins. For example, immediately after their synthesis at ribosomes, histone H3 interacts with the heat shock chaperone HSC70 (5,6), whereas histone H4 interacts with HSP90/HSP70 and the proteins PP32/Set (5,8). The association with HSP90 and tNASP unite histones H3 and H4 via the histone folding domain, thus allowing histones H3 and H4 to dimerize for the first time. Given that H3 and H4 associate together in this complex and the importance of H3–H4 dimers for chromatin assembly, we hypothesize the existence of a folding quality control step acting at this stage. How post-translational quality control of the histone folding process proceeds on newly synthesized histones remains unknown. In contrast, existing evidence suggests the importance of regulating histone protein levels as histone insufficiency or excess is harmful to the cell (11,12). Buffering of the soluble histone excess by the histone chaperone ASF1 (13) and histone degradation via ubiquitination and the proteasome (14) are some mechanisms that protect the cell from changes in histone levels. In yeast, excess non-chromatin associated histones is degraded with a half-life of around 30–40 min (15). In mammals, the soluble pool of H3 and H4 is buffered in a reservoir determined by association with the histone chaperone NASP (16). In response to NASP depletion, the stability of soluble pool of histones H3 and H4 decreases and is degraded independently of proteasome activity, but in a form that is dependent on the expression of LAMP2A (16), the receptor for chaperone-mediated autophagy (CMA). However, whether histones H3 and H4 are directly degraded by this lysosomal pathway remains unknown and a role for CMA in histone quality control still needs to be identified.

Here, we explore the molecular mechanisms contributing to the quality control of newly synthesized histone H3 and H4. By designing a mutant histone H3 that is not correctly folded and is unable to dimerize with histone H4, we uncover the molecular mechanisms set by cells for the degradation of unfolded histone H3. Consistent with a previous report (16), we found that histones H3 and H4 are *bona fide* substrates of CMA and that cytosolic histones H3 and H4 are mainly degraded by the CMA-dependent lysosomal pathway. We further investigated how histone degradation is regulated and found an interplay between HSC70 and tNASP, where they compete each other for the binding to H3, HSC70 targets histone H3 to lysosomes while tNASP protects it from degradation. This balance between two chaperones and their divergent outcomes is key for understanding how cells cope with defective newly synthesized histones and may be exploited in diseases affecting either degradation pathways or histone folding.

## MATERIALS AND METHODS

### Antibodies

The following antibodies were used in this study: β-actin (Sigma-Aldrich, A5441), ASF1 and NASP (from Genevieve Almouzni), Calreticulin (Cell Signaling Technologies, 2891), FLAG (Sigma, F1804), HIRA (Abcam, ab20655), Histone H2A (Abcam, ab18255), Histone H3 (Abcam, ab1791), Histone H4 (Abcam, ab10158), HSC70 (Abcam, 19136), HSP90 (Abcam, ab13492), Importin 5 (Santa Cruz, sc11369), GAPDH (Santa Cruz Biotech, 365062), LAMP1 (Abcam, ab25630), LAMP2A (ThermoFisher Scientific, 512200), LC3B (Cell Signaling, 2775), TopoI (Santa Cruz, sc32736), VDAC (Abcam, ab15895), Alexa 488 goat anti-mouse IgG (H + L) (ThermoFisher Scientific, A11001), anti-Mouse IgG HRP (Rockland, 610–1302), anti-Rabbit IgG HRP (Rockland, 611–1322), anti-Rat IgG HRP (Rockland, 612-703-002).

### Plasmids

The following plasmids were used in this study: HaloTag Histone H3 and H4 (GenScript, Piscataway, NJ, USA), His-H3A95P pET28a+ (made here), HaloTag-H3A95P pET28a+ (made here), His-H3 pET28a+, His-H3 Mut1 pET28a+ (made here), His-H3 Mut2 pET28a+ (made here), His-H3 DM pET28a+ (made here), HaloTag-H3 Mut1 pET28a+ (made here), HaloTag-H3 Mut2 pET28a+ (made here), HaloTag-H3 DM pET28a+ (made here), His-tNASP pET30 (Eric Campos), NASP-FLAG pCMV5 (MRC PPU Reagents and services).

### Cell culture and transfection

HeLa cells were obtained from ATCC and cultured in Dulbecco's modified Eagle's medium (DMEM) (Gibco) supplemented with 10% fetal bovine serum (Biological Industries). Lipofectamine 2000 (ThermoFisher Scientific) was used for cell transfections, according to the manufacturer′s instructions. For NASP knock-down, either 30 nM of negative control siRNA (Ambion, AM4611) or siRNA against NASP (Santa Cruz Biotechnology, sc-78745) were transfected into HeLa cells for 48 h.

### Measurement of cytosolic H3 turnover

HeLa cells were treated for 10 min with the following inhibitors of protein degradation: 10 μM Lactacystin (proteasome inhibitor), 10 mM 3-methyl-adenine (3-MA, macroautophagy inhibitor) and 20 mM ammonium chloride plus 200 μM leupeptin hemisulfate (lysosome inhibitor). Then, cells were incubated with 100 μg/ml cycloheximide in the presence of the inhibitors of protein degradation as indicated. After treatment, HeLa cells were washed with ice-cold PBS, scrapped, and pelleted at 350 g for 5 min. Cytosolic extracts were prepared by resuspending the cell pellet in hypotonic lysis buffer (5 mM potassium acetate, 0.5 mM MgCl_2_ and 20 mM HEPES, pH 7.4) using 10 strokes with the type B Dounce homogenizer. Homogenates were ultracentrifuged at 100 000 g for 1 h at 4°C. Supernatants were collected and proteins quantified using the BCA Protein Assay Kit (ThermoFisher Scientific).

### Cloning

The cDNA sequence of histone H3 (NP_003520.1) was synthesized (GenScript, Piscataway, NJ, USA) and fused to HaloTag using the pFN21A HaloTag^®^ CMV Flexi® vector (Promega) configured to append the HaloTag^®^ tag to the N-terminus of histone H3. To obtain His-H3 recombinant protein from bacteria, histone H3 cDNA (NP_003520.1) was subcloned (GenScript, Piscataway, NJ, USA) into pET28a+ plasmid (Novagen) to add a His-Tag on the N-terminus of histone H3. Histone H3A95P mutants were generated using the GENEART site-directed mutagenesis system (Invitrogen) and confirmed by sequencing. Mutants H3Mut1 (^52^IRRYQ^56^ to ^52^IRAAA^56^) and H3Mut2 (^82^DLRFQ^86^ to ^82^DLAAA^86^), both in pFN21A HaloTag® CMV Flexi^®^ vector or pET28a+ plasmid, were generated using the QuickChange II site-directed mutagenesis kit (Agilent) following the manufacturer instructions and confirmed by sequencing. Primers for H3Mut1: Forward: 5′cgctctccgcgagatccgcgccgccgcgaaatccaccgagctgctc3′, Reverse: 5′gagcagctcggtggatttcgcggcggcgcggatctcgcggagagcg3′; H3Mut2: Forward: 5′aggacttcaagaccgacctggccgccgcgagctcggccgttatggctc3′, Reverse: 5′gagccataacggccgagctcgcggcggccaggtcggtcttgaagtcct3′. To obtain H3DM (^52^IRAAA^56^ and ^82^DLAAA^86^ mutations), the set of primers for H3Mut2 were used over the vectors harboring H3Mut1. To express and purify recombinant His-HSC70 from bacteria, the coding sequence of human HSC70 (Addgene # 19487) was sub-cloned into the pET28a+ backbone (Novagen) by PCR amplification (Forward: 5′gatcgatcgatcgatccatatgtccaagggacctgcagttgg3′ and Reverse: 5′gatcgatcgatcgatcctcgagttaatcaacctcttcaatggtgg3′) and inserted between the NdeI and XhoI sites.

### Immunofluorescence

HeLa cells were seeded on 12 mm coverslips pretreated with 0.1 mg/ml poly-l-lysine and transfected with plasmids containing HaloTag-H3, HaloTag-H4, HaloTag-H3A95P, HaloTag-H3Mut1, HaloTag-H3Mut2, HaloTag-H3DM. After 24 h, cells were incubated with HaloTag-TMR ligand as indicated by the manufacturer′s instructions (Promega). Briefly, cells were incubated with 1 μM of HaloTag-TMR ligand for 15 min as a pulse, washed three times with PBS, incubated at 37°C for 45 min, and fixed with 4% paraformaldehyde at RT for 20 min. Cells were then permeabilized with 100 μg/ml digitonin, blocked with 0.02% gelatin and immunostained overnight at 4°C with antibodies against LAMP1. Cells were then stained with Alexa 488-conjugated secondary antibody and DAPI at 37°C for 30 min.

### Microscopy and image analysis

Microscopy images were acquired using an OLYMPUS FV1200 inverted confocal microscope with a 60× oil immersion objective (NA 1.4). For the analysis of lysosome location and colocalization, Z stack images of cells were captured in each channel at an interplane distance of 400 nm at optimal confocal apertures. Colocalization of H3 or H4 signal with lysosomes (LAMP1) was made in maximal projections of image stacks. First, the area of the nucleus in cells was bound by the thresholding DAPI signal and then subtracted from H3-HaloTag-TMR (H4-HaloTag-TMR) and LAMP1 image channels. Then, colocalization between the cytoplasmic HT-H3 (or HT-H4) signal and LAMP1 signal in these images was estimated using the ICA (Intensity Correlation Analysis) plugin of ImageJ software (17). Intensity Correlation Quotient (ICQ) values obtained were distributed between −0.5 and +0.5; representing random staining when ICQ ∼0; segregated staining when 0 > ICQ > −0.5; and dependent staining when 0 < ICQ < 0.5. Finally, potential differences between ICQ values of each treatment were assayed by Student's *t*-test.

### Expression of recombinant proteins

Histones H3, H3A95P, H3Mut1, H3Mut2, H3DM and H4; His-tNASP, His-sNASP and His-HSC70 were overexpressed in bacteria by inducing their expression with 0.2 mM IPTG for 2 h. Histones were purified from inclusion bodies, as previously described (18). tNASP and HSC70 were purified by Ni^2^^+^-agarose beads (Pierce), according to the manufacturer′s instructions. Purified GAPDH from rabbit muscle was purchased from Sigma-Aldrich.

### Lysosomal isolation

Sprague-Dawley rats were starved 48 h and euthanized by CO_2_ inhalation. Livers were removed, kept at 4°C in 0.25 M sucrose pH 7.2 and homogenized using seven strokes in a plotter homogenizer. Lysosomal fractions were isolated by ultracentrifugation through a discontinuous 85.6% Nycodenz (Accurate Biochemical) gradient at 141 000 g for 70 min, as previously described (19). Lysosomal fractions were collected, washed to remove Nycodenz excess, gently resuspended in 300 μl of 0.25 M Sucrose in 10 mM MOPS pH 7.3 and used immediately in lysosomal uptake assays. Lysosomal integrity was determined measuring the latent activity of β-hexosaminidase (β-Hex) using a fluorescent substrate. In short, for total β-Hex activity determination a 50 μl aliquot was lysed with 0.1% Triton X-100 and subjected to 10 cycles of freezing and thawing. To determine the activity outside lysosomes derived from broken or permeabilized organelles, a 50 μl sample was centrifuged at 15 000 rpm for 10 min and the supernatant was assayed for β-Hex activity. Percent of latency was determined by the difference between total and external β-Hex activity normalized by total activity.

### Functional lysosomal uptake assay and saturation curves

Sixty μg of intact and freshly isolated lysosomes with latency over 80% were resuspended in MOPS buffer (10 mM MOPS pH 7.3, 0.25 M sucrose), treated (+PI) or not (–PI) with a protease inhibitor cocktail (0.25 mM EDTA, 0.06 mM Leupeptin, 0.068 mg/ml Pepstatin A, and 0.14 mM PMSF) and incubated with 60 ng (4 pmol) of recombinant histone H3 (wild type or mutants) or 60 ng (4 pmol) of recombinant histone H4 at 37°C for 20 min, followed by centrifugation at 17 000 g for 20 min. The supernatant was removed, and the pellet was resuspended in 30 μl of MOPS buffer and analyzed by western blot. To properly compare the uptake between CMA– and CMA+ fractions, we determined LAMP2A levels in each fraction by western blot to normalize the experiment. In competition assays, 50, 100 or 200 ng tNASP (0.6–2.4 pmol) or sNASP (1–4 pmol) were pre-incubated with 60 ng of H3 or H4, followed by incubation with lysosomes, as described. Uptake of the CMA substrate into the lysosomal lumen was quantified by subtraction of the amount of protein associated with lysosomes in the absence of PI (binding) from the total amount of protein associated with lysosomal membranes and not degraded in the lysosomal lumen by the presence of PI (association), as determined in parallel assays (20). Saturation curves were obtained from –PI data after 20 min of incubation of isolated lysosomes with purified recombinant substrates. In these conditions substrate translocation to lysosomes is active and degradation of substrates by lysosomal proteases persist. Saturation indicates the maximal amount of total substrate in lysosomes, consisting of substrate bound to the surface, substrate in the process of translocation and substrate translocated but still not degraded after 20 min of incubation. However, as binding is probably the major substrate population in this fraction and protein translocation through LAMP2A pores is the limited step in CMA mechanism, calculated *K*_d_ represents most likely the binding affinity of substrates to lysosomal surfaces, mediated by LAMP2A receptor.

### Recombinant histone H3 and HSC70 interaction

Six hundred pmol (10 μg) of recombinant H3WT, H3Mut1, H3Mut2 or H3DM were incubated with 600 pmol (40 μg) of recombinant HSC70 in a buffer containing 10 mM Tris–HCl pH 7.5, 150 mM NaCl, 0.4 mM EDTA, 0.05% NP-40, 0.5 mM DTT, 0.2 mM PMSF for 30 min on ice. Then, the protein mixture was loaded onto a 5-ml 5–20% glycerol gradient in the same buffer. The gradient was ultracentrifuged at 26 000 rpm in a SW 55 Ti rotor for 16 h at 4°C. The gradient was fractionated in 250 μl aliquots and analyzed by western blot.

### Competition assay between recombinant HSC70 and tNASP for H3

Different molar combinations of the recombinant proteins, as indicated, were incubated in 500 μl of BC50 Buffer (20 mM Tris–HCl pH 7.5, 50 mM KCl, 0.2 mM EDTA, 10 mM 2-mercaptoethanol, 0.2 mM PMSF and 10% glycerol) supplemented with 0.05% NP-40, for 20 min on ice. Then, the protein mixture was loaded onto a Superdex 200 10/300 GL column (GE healthcare, 175175–01) equilibrated with BC50 containing 0.05% NP-40. The void volume, corresponding to 7 ml, as measured by the elution of Dextran-blue, was received in one tube and then fractions of 500 μl were collected. Aliquots derived from the fractions were analyzed by western blot.

### Design of unfolded H3 mutant

A mutation that disrupts the α-2 Helix of H3 protein was designed by *in silico* analysis using the GOR4 software, an algorithm which examines the proneness for each amino acid residue to form a complex structure with the surrounding residues. All alanine residues present in the α-2 Helix of H3 protein were changed to proline and analyzed one at a time. From the output, as a likelihood percentage, potential candidates were selected as the lowest percentage able to form an α-Helix structure (https://npsa-prabi.ibcp.fr/cgi-bin/npsa_automat.pl?page=/NPSA/npsa_gor4.html). From this analysis we selected the mutation H3A95P.

### Folding of recombinant H3–H4 tetramers

Two nmol of histone H4 (∼88 μg) and 2 nmol of either wild type H3 or H3A95P (∼96 μg) were mixed in unfolding buffer (7 M guanidinium HCl, 20 mM Tris–HCl pH 7.5, 10 mM DTT) and incubated for 1 h at 4°C. Samples were then dialyzed against refolding buffer (2 M NaCl, 10 mM Tris–HCl pH 7.5, 1 mM Na-EDTA, 5 mM 2-mercaptoethanol) and then centrifuged at 17 000 rpm for 20 min at 4°C. The supernatant containing H3–H4 tetramers was loaded onto a Superdex 75 10/300 GL column (GE Healthcare, 17-5174-01) equilibrated with refolding buffer, and 250 μl fractions were collected. Aliquots derived from the fractions were analyzed by Coomassie blue stained gels.

### Statistical analysis

For all western blot experiments quantified by densitometry, the statistical significance was obtained in Graphpad prism software. The *P* values were calculated using an unpaired Student's *t*-test, the error bars expressed as mean ± standard error of mean and the sample size (*n*) indicated in figure legends.

## RESULTS

### Newly synthesized histones H3 and H4 are degraded by lysosomes

**Figure 1.**
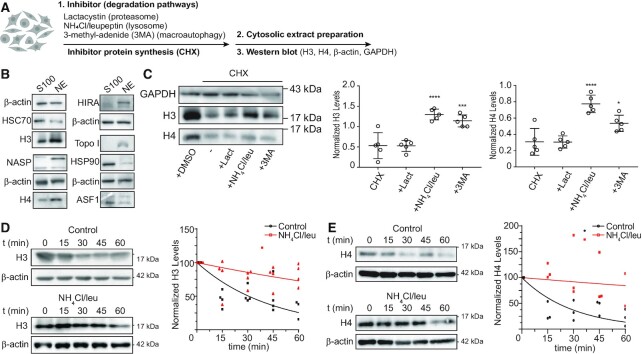
Newly synthesized histones H3 and H4 are degraded by lysosomes. (**A**) Scheme illustrating the experimental approach to assessing cytosolic H3 and H4 levels after the inhibition of different protein degradation pathways on HeLa cells. Cells were pre-incubated for 10 min with 10 μM Lactacystin, 20 mM NH_4_Cl + 200 μM leupeptin, and 10 mM 3-MA, followed by 1 h treatment with 100 μg/ml CHX in the presence of the inhibitors. (**B**) Western blot analyses of 5 μg of cytosolic (S100) and nuclear (NE) extracts derived from HeLa cells. (**C**) Left, representative immunoblot of 5 μg of cytosolic extract. Graph shows normalized levels of cytosolic H3 (left) and H4 (right) by GAPDH signal on each condition. *N* = 5 independent experiments; *P-value < 0.05, ***P-value < 0.001, ****P-value < 0.0001, Student's *t*-test. (**D**, **E**) Left, HeLa cells were pre-incubated with or without 20 mM NH_4_Cl + 200 μM leupeptin for 10 min before addition of 100 μg/ml CHX. Cytosolic extracts were prepared at 0, 15, 30, 45 and 60 min after treatment and analyzed by western blot for H3 (D) and H4 (E). Right, histone H3 (D) and H4 (E) signal was quantified by densitometry and normalized against β-actin. Mean values obtained from 4 (H3) and 3 (H4) independent experiments were adjusted to a non-linear regression (first-order exponential decay).

To investigate whether newly synthesized histones H3 and H4 are degraded as part of their quality control mechanism in the cytosolic maturation pathway, we determined H3 and H4 protein levels in cytosolic extracts following inhibition of different degradation pathways. For this, we pre-incubated HeLa cells with Lactacystin to inhibit the proteasome, ammonium chloride plus leupeptin to inhibit whole lysosomal-dependent degradation, or 3-methyl-adenide (3-MA) to inhibit macroautophagy. We then blocked protein synthesis with cycloheximide (CHX) and followed H3 and H4 protein levels in cytosolic extracts by western blot (Figure 1A). We tested the quality of the extracts by western blot observing that HSC70, HSP90 and ASF1a/b were enriched in cytosolic fractions, whereas histones H3 and H4, tNASP, HIRA and Topoisomerase I in nuclear extract (Figure 1B). The inhibition of protein degradation pathways did not affect neither cell cycle (Supplementary Figure S1A) nor the protein levels of components of the histones H3/H4 nuclear translocation machinery, including Importin 5 and ASF1a/b (Supplementary Figure S1B). The treatment did not affect the histone H3/H4 cytoplasmic to nuclear ratio either (Supplementary Figure S1B). We previously found that cytosolic H3 and H4 pools mainly represent newly synthesized histones (8,21). Therefore, we considered cytosolic histones as newly synthesized pools. As indicated by the levels of cytosolic histone H3, remaining histone levels after inhibition of protein synthesis are not affected when inhibiting the proteasome. In contrast, upon lysosomal and macroautophagy inhibition, the levels of the stable pool of cytosolic H3 increases by 2.5 and 2.2 times, respectively (Figure 1C). Similarly, cytosolic histone H4 levels are not affected by inhibiting the proteasome, whereas its remnant levels after protein synthesis inhibition increases by 2.5 and 1.7 times upon lysosomal and macroautophagy inhibition, respectively (Figure 1C). However, given that lysosomal and macroautophagy inhibition affect proteins globally, including GAPDH, one cannot rule out that the effect on histones might be pronounced by normalizing against GAPDH. Thus, in the following experiments we normalized against β-actin. We then determined that under our conditions, the half-life of histone H3 in the cytosol is 30.51 min (Figure 1D). Half-life of newly synthesized histone H3 in the cytosol is short as it quickly translocates to the nucleus for chromatin assembly (22). This half-life increases to 130.8 min upon treatment of cells with ammonium chloride/leupeptin (Figure 1D), confirming the importance of lysosomal degradation in regulating the cytosolic levels of newly synthetized H3. Similarly, the half-life of histone H4 in the cytosol is 20.85 min, which increases to 243.2 min following the treatment of cells with ammonium chloride/leupeptin (Figure 1E). Inhibition of lysosomal degradation with ammonium chloride/leupeptin is not specific for lysosomal histone degradation, but the magnitude of change in their half-life of >10-fold for both H3 and H4 strongly suggests the involvement of the lysosome in the control of cytosolic histone levels.

### Newly synthesized histones H3 and H4 are targeted to lysosomes

**Figure 2.**
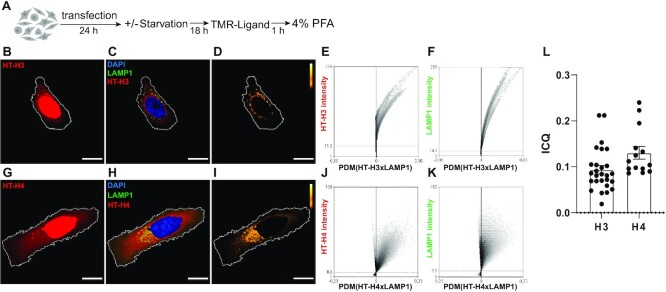
Newly synthesized histones H3 and H4 are targeted to lysosomes. (**A**) Scheme illustrating the experimental approach to evaluate the targeting of histones H3 and H4 to the lysosomal compartment by microscopy imaging. (**B, C, D, G, H, I**) Representative confocal microscopy images of HeLa cells expressing HaloTag-H3 (HT-H3) (B–D) and HaloTag-H4 (HT-H4) (G–I) and stained with HaloTag-TMR ligand (red), DAPI (blue) and immunostained against LAMP1 (green). (B, G) Images of HaloTag-TMR staining showing the distribution of HT-H3 (B) and HT-H4 (G) (red) in cells. (C, H) merge images of HaloTag-TMR staining (red), DAPI (blue) and LAMP1 (green) for H3 (C) and H4 (H). (D, I) Pseudocolored images showing the pixels that colocalize (yellow/white) or show exclusion (black) for the staining between HaloTag-TMR and LAMP1 in HeLa cells, according to ICA analysis, for H3 (D) and H4 (I). The area of nuclei corresponding to DAPI staining was subtracted from images of HaloTag-TMR (red) and LAMP1 (green) channels and for colocalization analysis. Scale bar = 10 μm. (E, F, J, K) Representative ICA graphs obtained from the intensity correlation analysis of cells expressing HT-H3 (E, F) and HT-H4 (J, K), and immunostained with LAMP1 24 h post-transfection, where the Y-axis corresponds to a scale for 8-bit images ranging from 0 to 256, and the X-axis corresponds the covariance of the mean intensity (PDM = product of the differences from the mean). (**L**) Intensity Correlation Quotient (ICQ) graph, as HT/LAMP1 signal of 27 (HT-H3) and 14 (HT-H4) cells for condition.

We next evaluated the direct targeting of histones H3 and H4 into the lysosomal compartment by immunofluorescence and microscopy image analysis. We expressed human H3 and H4 proteins fused to a HaloTag (HT) peptide into their N-terminal tails (23) and visually inspected their location by confocal fluorescence microscopy. HT is a 297-residue peptide (33 kDa) derived from a bacterial enzyme, with a dehalogenase activity that covalently binds chloroalkane fluorescent HT-ligands only at a neutral pH. This allows proteins to be marked with permeable fluorescent probes outside acidic organelles and then to follow their traffic and redistribution through the cell and organelles, including lysosomes (24). HeLa cells transfected with either HaloTag-H3 or HaloTag-H4 constructs (HT-H3 or HT-H4) were maintained for 24 hours as illustrated in Figure 2A. As shown in Figure 2B and G, after staining and 1 hour of probe washout, most of the HT-H3 and HT-H4 fluorescence signals were in the cellular nuclei, as expected, with some of the staining occupying cytoplasmic perinuclear areas in discrete structures. Partial colocalization and positive spatial intensity correlations of HT-H3 and HT-H4 staining with lysosomes, labeled with LAMP1, were observed in all HT-H3/HT-H4 expressing cells, suggesting a rapid translocation of H3 and H4 into lysosomes (Figure 2C, H, and Supplementary Figure S2). A portion of newly synthesized histones H3 and H4 were enriched in lysosomes as observed by images of positive product difference of the mean (PDM) pixel intensities of HT-H3 or HT-H4 and lysosome image channels (Figure 2D and I and Graphs 2E–2F and 2J–2K) indicating positive intensity correlation and partial colocalization. Colocalization was quantified using Intensity Correlation Quotients (ICQ) (see Materials and Methods), resulting in ICQ = 0.09 ± 0.13 for H3 and LAMP1 and ICQ = 0.13 ± 0.05 for H4 and LAMP1 (Figure 2L).

### Recombinant histones H3 and H4 are *in vitro* substrates of CMA

**Figure 3.**
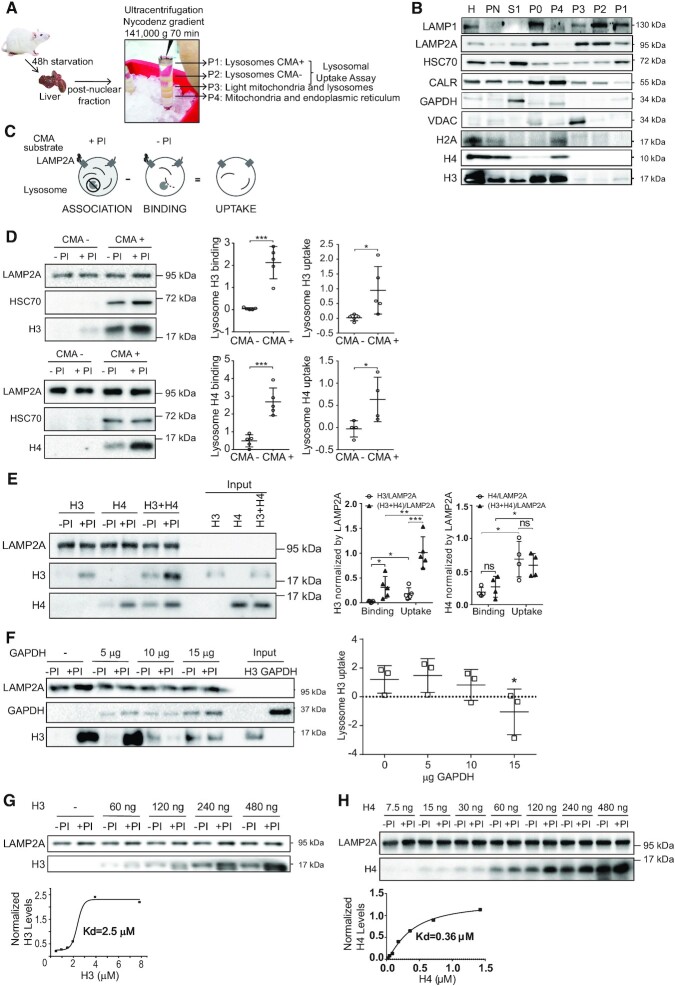
Histones H3 and H4 are degraded by CMA *in vitro*. (**A**) Scheme of lysosome extraction from the rat liver. (**B**) Immunoblot analyses loading 30 μg of the different fractions collected, H: liver homogenate, PN: post-nuclear fraction, S1: supernatant of PN, P0: input of the endoplasmic reticulum, mitochondria and lysosomal fractions, P1: lysosomes enrichment in the CMA activity (CMA+), P2: lysosomes mix of CMA+ lysosomes and those with low CMA activity (CMA–), P3: lysosomes and light mitochondria, and P4: mitochondria and endoplasmic reticulum. (**C**) Experimental scheme to investigate lysosomal association, binding and uptake of target substrates, in the presence (+) or absence (−) of protease inhibitors (PI). (**D**) Left, representative immunoblot of the uptake assay using 60 μg of lysosomes CMA– and CMA+ fractions with histones H3 (top) and H4 (bottom). Right, graphs of binding and uptake, quantifying the signal of histone H3 (top) and H4 (bottom) normalized by the LAMP2A signal from five independent experiments: ***P < 0.001, *P< 0.05, Student′s *t*-test. (**E**) Left, immunoblot of the uptake assay, comparing binding and association of 60 ng of recombinant histone H3, 60 ng of recombinant histone H4, or 60 ng of recombinant histone H3 and 60 ng of H4. For uptake assays with both substrates, aliquots of recombinant H3 and H4 were mixed at 4°C and immediately added to lysosomes. Input represents 10% of the reaction. Right, graphs of binding and uptake, quantifying the signal of histone H3 and H4 normalized by the LAMP2A signal from five (histone H3) and four (histone H4) independent experiments: ***P < 0.0001, **P < 0.001, *P< 0.005, Student′s *t*-test. (**F**) Left, representative immunoblot of the uptake assay of 60 ng of histone H3, upon increasing amounts of recombinant GAPDH. Input represents 10% of the reaction. Right, graph of the uptake assay, quantifying the signal of histone H3 and GAPDH normalized by the LAMP2A signal from three independent experiments: *P < 0.05, Student's *t*-test. (G, H) Top, representative immunoblot of the uptake assay with increasing concentrations of recombinant histone H3 (**G**) or histone H4 (**H**). Bottom, saturation curve obtained from the quantification of the histone H3 (G) or histone H4 (H) signal from the western blot in the absence of protease inhibitors (–PI, binding) normalized against the LAMP2A signal, as explained in detail in Materials and Methods.

The direct measurement of CMA-mediated degradation of protein substrates can be performed by *in vitro* lysosomal uptake assays (25). To examine whether H3 and H4 are direct substrates of CMA, we isolated lysosomes from rat livers by subcellular fractionation followed by density gradient ultracentrifugation, as illustrated in Figure 3A. The quality of lysosomal purification was analyzed by western blot. We observed that lysosomal markers LAMP1 and LAMP2A were enriched in both, fractions P1 (CMA+) and P2 (CMA–). HSC70, the molecular chaperone that targets CMA substrates to lysosomes, was enriched in P1 (CMA+) compared to P2 (CMA–) fractions, whereas the mitochondria and endoplasmic reticulum markers VDAC and Calreticulin (CALR) were enriched in fractions P3 and P4, respectively (Figure 3B, Supplementary Figure S1C). Interestingly, we were able to detect endogenous histone H3 on the P1 (CMA+) fraction. Integrity of lysosomal fractions was verified measuring the activity of extra-lysosomal enzyme β-hexosaminidase in the CMA+ fractions (lysosomal latency, Supplementary Figure S1D). These results confirmed the integrity and quality of lysosomal fractions for CMA transport assays. We then performed the *in vitro* lysosomal uptake assay with recombinant histones H3 and H4, as illustrated in Figure 3C and previously described (20). Interestingly, we found that histones H3 and H4 are substrates of CMA (Figure 3D). CMA (+) lysosomes, reported to contain increased levels of LAMP2A and HSC70, were more efficient than CMA (−) lysosomes in the binding and uptake of H3 and H4 (Figure 3D). We then investigated whether the targeting of histones to lysosomes is regulated by the presence of their partner histone. For this, we performed the assay with H3, H4 and mixing H3 and H4 proteins at 4°C and immediately added to lysosomes. We did not use tetramers H3/H4 as they would not be stable at the salt concentration used in the assay. Lysosomal uptake of H4 was around 3-fold higher than H3. We found that the addition of H4 to H3 stimulates the binding and uptake of H3 to lysosomes, but the addition of H3 to H4 does not (Figure 3E). It should be noticed that the result of this experiment might be influenced using two distinct antibodies (against H3 and H4) that have different affinities for their substrates. To evaluate whether H3 uptake is facilitated by the same machinery that mediates the uptake of other known CMA substrates, we performed a lysosome uptake competition assay using recombinant histone H3 in the presence of increasing concentrations of recombinant GAPDH. As observed in Figure 3F, lysosomal uptake of histone H3 was competed with and inhibited by an excess of recombinant GAPDH. Finally, we found that recombinant histones had a saturable dose-response binding to purified rat liver lysosomes with a *K*_d_ of 2.5 μM for H3 (Figure 3G), and a *K*_d_ of 0.36 μM for H4 (Figure 3H). These numbers are similar to those reported for other CMA substrates such as GAPDH (*K*_d_ = 10 μM) (26). Together these results indicate that histones H3 and H4 are *bona fide* CMA substrates.

**Figure 4.**
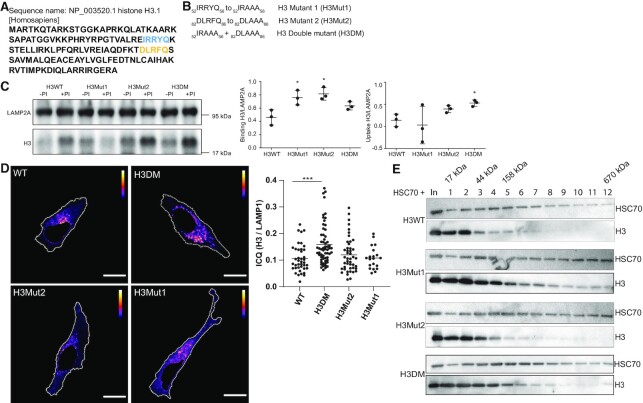
The KFERQ motifs on histone H3 play a negative role on the targeting to CMA. (**A**) Analysis of KFERQ sequences using KFERQ finder software (28) identify two motifs in H3.1 protein, a canonical ^82^DLRFQ^86^ motif (yellow) and a putative ^52^IRRYQ^56^ motif (blue). (**B**) Mutants of the histone H3 KFERQ motifs. (**C**) Left, representative immunoblot of the uptake assay using histone H3WT, H3Mut1, H3Mut2 and H3DM. Right, graphs of binding and uptake, quantifying the signal of histone H3 normalized by the LAMP2A signal from three independent experiments: *P< 0.05, Student′s *t*-test. (**D**) Left, representative pseudocolored images of HeLa cells expressing HaloTag-H3WT, HaloTag-H3Mut1, HaloTag-H3Mut2 and HaloTag-H3DM, showing the pixels that colocalize (yellow) or show exclusion (blue) for the staining between HaloTag-TMR and LAMP1 in HeLa cells, according to ICA analysis. The area of nuclei corresponding to DAPI staining was subtracted from images of HaloTag-TMR (red) and LAMP1 (green) channels and for colocalization analysis. Right, Intensity Correlation Quotient (ICQ) graph, as HT/LAMP1 signal for each of the mutants. Scale bar = 10 μm. (**E**) Interaction analysis between recombinant histone H3 and HSC70. Ten μg (600 pmol) of either recombinant H3WT, H3Mut1, H3Mut2 or H3DM were mixed with 40 μg (600 pmol) of recombinant HSC70 and resolved by a 5–20% glycerol gradient. Immunoblot of fractions derived from the gradient were analyzed as shown. Fraction number 1 corresponds to the lightest fraction collected. In: input, it represents 1% of the sample.

Chaperone-mediated autophagy (CMA) facilitates the direct translocation of selected protein substrates to lysosomes through membrane pores formed by the LAMP2A protein (20). The targeting mechanism relies on the recognition of specific protein substrates containing the amino acid sequence of a pentapeptide motif biochemically related to KFERQ by the molecular chaperone HSC70 (27). The analysis of KFERQ sequences using KFERQ finder software (28) identifies 2 motifs in the H3.1 protein; a canonical ^82^DLRFQ^86^ motif and a putative (potentially activated by phosphorylation) ^52^IRRYQ^56^ motif, that are conserved in the H3.1, H3.2 and H3.3 variants (Figure 4A). No KFERQ motifs were predicted in the histone H4 protein. To investigate if the KFERQ motifs are necessary for H3 mediated CMA degradation, we designed the following KFERQ histone H3 mutants: ^82^DLAAA^86^ (H3Mut1), ^52^IRAAA^56^ (H3Mut2), and the double mutant ^52^IRAAA^56^ + ^82^DLAAA^86^ (H3DM) (Figure 4B). None of the H3 mutants affected the formation of the tetramer with histone H4 (Supplementary Figure S1E). We analyzed the mutants on their cellular location by *in vitro* uptake assays and fluorescence (Figure 4C and D). Contrary to our expectations, single mutants had an increase binding and no effect on uptake, whereas the uptake was increased in the double mutant (Figure 4C). Immunofluorescence and microscopy image analysis showed that the double mutant colocalized more with lysosomes when compared to the wild-type histone H3 (Figure 4D and Supplementary Figure S3). We next investigated the interaction between histone H3 mutants and HSC70 by mixing equimolar amounts of histone H3 and HSC70 and resolving the association by glycerol gradient (Figure 4E). Western blot showed some association between H3 and HSC70 (fraction 2), which was more evident with H3 mutants, specially H3Mut1 (observed by the displacement of H3 toward heavier fractions (3–5 and beyond). Therefore, we concluded that ^82^DLRFQ^86^ and ^52^IRRYQ^56^ H3 motifs play a negative role on CMA-mediated degradation likely by regulating the binding of H3 to HSC70.

### tNASP and HSC70 compete for the binding to histone H3, regulating its targeting to lysosomes

**Figure 5.**
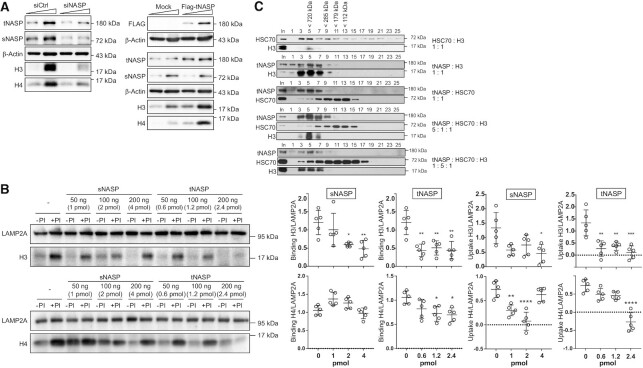
tNASP and HSC70 compete for the binding to histone H3 regulating its targeting to the lysosomes. (**A**) Immunoblot of cytosolic extract from HeLa cells untreated (siCtrl) or treated with either siNASP to deplete NASP mRNA levels, or Flag-tNASP to overexpress its levels, during 48 h. Ten and 20 μg of cytosolic extracts were analyzed by western blot. (**B**) Left, representative immunoblot of an uptake assay with either 60 ng of histone H3, corresponding to 4 pmol of protein (top), or 60 ng of histone H4 (bottom) incubated with increasing amounts of recombinant protein tNASP (0.6–2.4 pmol) or sNASP (1–4 pmol). Right, graph of binding and uptake, quantifying the signal of histone H3 (top) or H4 (bottom) normalized by the LAMP2A signal, from five independent experiments: ****P< 0.0001, ***P< 0.001, **P< 0.01, *P< 0.05, Student's *t*-test. (**C**) Interaction analysis between recombinant histone H3, HSC70 and tNASP at different molar ratios were resolved by the size exclusion chromatography column S200. Immunoblot of odd fractions derived from the column were analyzed as shown. Fraction number 1 corresponds to the first fraction collected after the elution of the void volume. In, input.

**Figure 6.**
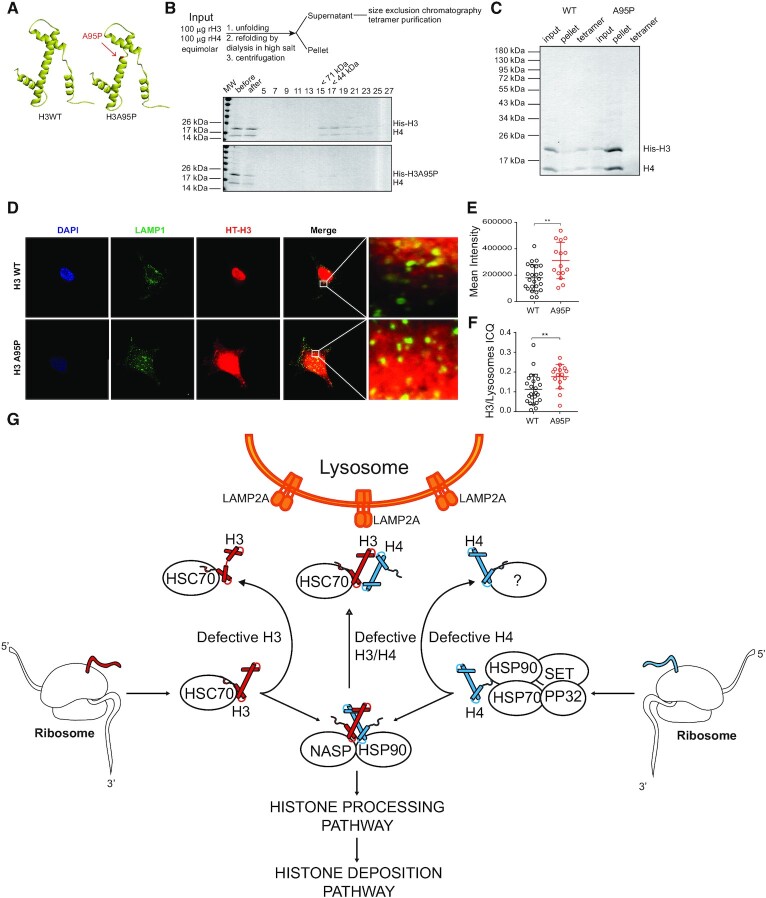
CMA participates in the degradation of misfolded histone H3 protein. (**A**) Ribbon diagram illustrating the structures of wild-type histone H3 and mutant histone H3A95P. The arrow points to the site where A95 is located along the second α-helix and the disruption of the helix upon mutation to proline. (**B**) Top, scheme illustrating the experimental procedure to assemble tetramers. Bottom, Coomassie blue staining analysis of fractions derived from the S200 sizing column after tetramer formation utilizing wild type His-tagged or His-H3A95P. (**C**) Coomassie blue staining gel of samples derived from the tetramer assembly reaction (input, pellet and tetramer derived from the S200 column) with wild type His-tagged and His-H3A95P. (D–F) Immunofluorescence of HeLa cells for LAMP1 (green), DAPI (blue), and HT-H3 or -H3A95P (red) (**D**). Mean intensity in arbitrary units of HT-H3 signal of 20 cells per condition (**E**). Intensity Correlation Quotient (ICQ) graph, as HT/LAMP1 signal of 20 cells per condition (**F**); **P< 0.01, Student′s *t*-test. (**G**) Model for the quality control of newly synthesized histones H3 and H4. The figure illustrates the relation between the biogenesis and the degradation mediated by Chaperone Mediated Autophagy (CMA) of histones H3 and H4 in the cytoplasm. As shown in this work, CMA participates in the degradation of histones H3 and H4 and possibly in the quality control of histones by degrading unfolded or defective newly synthesized histones H3 and H4. The chaperones HSC70 and tNASP are important regulators in this pathway, in such a way that HSC70 can either assist in the proper folding of histones or deliver H3 to the LAMP2A receptor at the lysosome for degradation. Whether histone H4 is also targeted by HSC70 to the lysosome is currently unknown. tNASP instead stabilizes and prevents histones H3 and H4 degradation.

We next explored how the CMA-mediated degradation of histones is regulated and whether an interplay between chaperones is in place. The histone chaperone NASP has two splice variants: tNASP, which is found in cancer, embryonic and germ cells, and sNASP, found in embryonic and somatic cells (29). For the maintenance of cytosolic steady-state levels of histone H3 in HeLa cells, tNASP/sNASP are necessary, as siRNA-mediated tNASP/sNASP knock-down in HeLa cells decreases the cytosolic levels of histone H3 (Figure 5A, (6,16)). In contrast, tNASP overexpression increases H3 levels (Figure 5A, (6,16)). However, the role of each NASP variant and the mechanism involved in NASP-mediated protection of the cytosolic histone H3 remains unclear. We hypothesize that NASP stabilizes histone H3 levels by blocking the direct transport of histones into lysosomes. To examine this, purified rat liver lysosomes were incubated with recombinant histones H3 (Figure 5B, top) and H4 (Figure 5B, bottom) and increasing amounts of either recombinant sNASP or recombinant tNASP. We observed that recombinant sNASP inhibited histone H3 lysosomal binding and uptake at molar ratios of 1:1 of sNASP:H3 (4 pmol, Figure 5B). sNASP only inhibited the uptake of histone H4. However, tNASP efficiently inhibited histone H3 lysosomal binding and uptake, starting at molar ratios of 1:7 of tNASP:H3 (0.6 pmol, Figure 5B). tNASP inhibited binding and uptake of histone H4, but at higher concentrations. We conclude that tNASP is more efficient than sNASP to inhibit histone lysosomal binding and uptake, and when compared H3 and H4, tNASP is more efficient to block H3. Given that histone H3 associates to HSC70 during its maturation pathway in the cytoplasm, we analyzed whether tNASP competes with HSC70 for the binding to histone H3. We mixed equimolar amounts of each protein and the resultant protein complexes were resolved by S200 size exclusion chromatography. Western blot of fractions derived from the S200 column showed that HSC70 interacts with histone H3 forming a complex of about 700 kDa (Figure 5C, fraction 5). The size of this complex is larger than the expected 250 kDa complex previously characterized (5,6), and we do not know the reason of this. A similar size complex is formed by tNASP and histone H3 (Figure 5C, fractions 3–7). tNASP and HSC70 do not coelute, indicating that they do not interact with each other (Figure 5C, tNASP peaks in fraction 5, while HSC70 in fractions 11–13). Interestingly, when a 5-fold molar excess of tNASP was added, the HSC70/H3 interaction was displaced, favoring tNASP/H3 association (Figure 5C, fraction 5). When HSC70 was added in excess, tNASP was still able to displace the interaction of HSC70/H3 (Figure 5C, fraction 5). Taken together, our results indicate that tNASP regulates the stabilization of cytosolic histone H3 pools, displacing HSC70/H3 binding and thus blocking the targeting of histone H3 to CMA.

### CMA participates in the degradation of misfolded histone H3 proteins

We then investigated whether CMA regulates the levels of misfolded H3 as part of a mechanism of protein quality control. To examine this, we designed a mutation in histone H3 that disrupts the interaction with histone H4. Given that H3-H4 interactions mainly occur across the α-2 helix of each protein (30), we mutated the alanine 95 to proline (H3A95P) to disrupt this helix (Figure 6A, red arrow). We bacterially expressed and purified wild-type His-tagged H3 (His-H3) and mutant alanine-to-proline, at position 95, His-tagged H3 (His-H3A95P) proteins. To confirm the loss of histone H3A95P and H4 association, we mixed equimolar amounts of recombinant histones H4 and either wild type His-H3 or His-H3A95P under denaturing conditions and induced the folding of H3–H4 into a tetramer (H3–H4)_2_ by high salt dialysis (Figure 6B, top). The sample was then centrifuged to remove any precipitates and soluble proteins were resolved by S200 size exclusion chromatography (Figure 6B, bottom). We observed the peak of (H3–H4)_2_ tetramer elution with His-tagged H3 on fractions 17–19, corresponding to a molecular size of about 50 kDa (Figure 6B). In contrast, we could not observe proteins eluting on the sizing column from samples containing H3A95P (Figure 6B). We then examined input, pellet and tetramer fractions on Coomassie blue-stained gels and observed that, while most of the wild type His-tagged H3 formed tetramers, the majority of H3A95P was found in the pellet fraction (Figure 6C). Thus, we conclude that the mutant H3A95P is unable to interact with histone H4. To analyze the targeting efficiency of misfolded histone H3 to lysosomes, we analyzed the colocalization of HaloTag-fused wild-type H3 and H3A95P with the lysosomal marker LAMP1 in HeLa cells. As observed in Figure 6D, both cytosolic wild type H3 and H3A95P partially colocalized with lysosomes. Interestingly, the intensity of cytosolic H3A95P was significantly higher than wild-type H3 (Figure 6E). Quantification of the histone H3 colocalization with lysosomes by ICQ analysis indicated that H3A95P significantly increased its interaction with the lysosomal compartment compared to the wild-type H3 protein (Figure 6F), suggesting that misfolded H3 is efficiently transported to lysosomes.

## DISCUSSION

Ensuring the supply of properly processed high-quality histones toward the nucleus for chromatin assembly is essential for the preservation of genome integrity and function. In this work, we show biochemical evidence that chaperone-mediated autophagy (CMA) is the main degradation pathway of newly synthesized cytosolic histones H3 and H4. This degradation is regulated by a fine-tuned interplay between the tNASP-mediated stabilization of histones, and the HSC70-mediated targeting of H3 to lysosomes for degradation. Moreover, we further demonstrate that misfolded cytosolic histone H3 is targeted to lysosomes for degradation. Taking the data together, we propose a model in which CMA is a key component of the quality control of newly synthesized histones H3 and H4 and the balance between a network of chaperones and their different outcome is key for understanding how cells cope with defective newly synthesized histones H3 and H4 (Figure 6G). Recent reports showed that the chaperone DNAJC9 binds to histones H3 and H4 and recruits heat shock chaperones to fold histone dimers during the maturation cascade of newly synthesized histones and at the time of their deposition into chromatin (10,31). Considering our results, we hypothesized that if the folding activity mediated by the heat shock proteins network fails, defective newly synthesized histones H3 and H4 are degraded by CMA. To better understand the interplay between folding and degradation of histones it will be important to investigate the relationship between DNAJC9 and the HSC70/tNASP chaperones. In this context, it would be interesting to examine whether the histone chaperone ASF1, which interacts with sNASP, has any role in the CMA-mediated histone degradation. Finally, we suggest that this CMA-dependent quality control occurs early in the maturation cascade of newly synthesized histones, at the time in which histones H3 and H4 interact with each other and form the dimer. Histones H3/H4 that pass this quality control would continue their maturation cascade to finally associate with sNASP/RbAp48/Asf1/Importin for their nuclear translocation (5,6).

In eukaryotic cells, the co-translational ubiquitination of nascent misfolded polypeptides is the main quality control mechanism of newly synthetized proteins (32,33). In fact, about 30% of newly synthesized proteins are ubiquitinated at the ribosomes and degraded by the proteasome (32,33). Our analyses demonstrated that the proteasome activity could not explain cytosolic H3 and H4 proteolysis, whereas lysosomal degradation is the main cytosolic H3 and H4 degradation pathway (Figure 1A). It was assumed that CMA-degraded proteins partially unfold, making KFERQ-like motifs accessible to HSC70 for targeting to CMA. However, the evidence suggests that several proteins have solvent-exposed KFERQ motifs and that CMA participates in the continual removal of functional proteins, mainly in response to nutrient deprivation (28,34). We identified two KFERQ motifs in histone H3 and none on histone H4. However, contrary to our expectations, mutation of these motifs stimulated the binding and uptake of histone H3 to lysosomes and the colocalization of histone H3 with lysosomes in cells. In this work, we have confirmed that tNASP inhibits the binding and translocation of H3 and H4 to the lysosome and at least in the case of H3 is able to displace its binding to HSC70, due to its higher affinity. This suggests that tNASP binds to the KFERQ motifs of H3, displacing the histone interaction with HSC70. If the KFERQ mutant forms of H3 cannot be stabilized by binding to tNASP, then H3 could form an instable pool available for CMA degradation, explaining why increased degradation is observed in the KFERQ mutants H3 assayed in this work. Additional experiments are needed to test this hypothesis. On the other hand, there is no evidence of KFERQ motifs negatively affecting CMA-dependent degradation of protein substrates. However, post-translational modifications like ubiquitination and acetylation of KFERQ motifs of some substrates are a required step for CMA targeting (35–37). Further studies will be required to characterize the HSC70 targeting motif to lysosomes on histones H3 and H4.

An important aspect to investigate is whether the CMA-mediated histone H3 and H4 degradation is restricted to a quality control step in histone biogenesis, as shown in here, or whether it can also participate in the regulation of the excess of cytosolic histones. So far, the regulation of histone excess has been linked to proteasomal degradation (14). In either case, the control of histone supply to chromatin might have important effects in cell cycle progression and genome stability. Lysosomes, similar to histones, are essential for cell-cycle progression, genome integrity, and chromosomal stability (38–41). Deficiency in autophagy-dependent lysosomal degradation induces DNA damage and chromosomal instability (42). Thus, many questions remain unanswered. For example, could changes in CMA activity regulate cell cycle through the control of cytosolic histone levels? Does CMA activity or accessibility to histones H3 and H4 change during the cell cycle? We performed our experiments in asynchronous HeLa cells; thus, it would be interesting to explore H3/H4 degradation throughout the cell cycle.

DNA damage-induced malignancy transformation is related to a decrease in both macroautophagy and CMA activities (43), which can cause the mis-regulation of proteins like CHK1, making the cells hypersensitive to genotoxic insults (43). Additionally, increased levels of circulating histones have been observed in several diseases, including cancer and inflammation (44). It would be worth to further investigate how misfolded histones could be a relevant factor in the progression of different diseases, and whether there is a relationship with CMA activity, especially in cancer.

## DATA AVAILABILITY

All data are available from the corresponding authors upon request.

## Supplementary Material

gkab1296_Supplemental_File

## Notes

Present address: Juan Hormazabal, Instituto de Ciencias e Innovación en Medicina, Facultad de Medicina Clínica Alemana Universidad del Desarrollo, Santiago, Chile.
