# Supplementary material for: Chaperone mediated autophagy contributes to the newly synthesized histones H3 and H4 quality control
Source: Nucleic Acids Res. 2022 Jan 17;50(4):1875–87. doi: 10.1093/nar/gkab1296 (PMC8887419; doi:10.1093/nar/gkab1296)
Supplement: gkab1296_Supplemental_File [file gkab1296_supplemental_file.docx]

**SUPPLEMENTAL MATERIAL**

**Cell cycle analysis**

Treated HeLa cells were fixed with 70% cold ethanol and stored overnight at -20 °C. Cells were then washed with PBS and incubated with 0.5 mL 40 µg/mL RNase A for 1 h at 37 °C. DNA was then stained adding 2 µL of 1 mg/mL propidium iodide directly into the samples, immediately before analysis. Samples were analyzed by flow cytometry with BD FACSDIVA^TM^ Software (BD Bioscience), and cell cycle examined with FlowJovX.0.7 (tree Star).


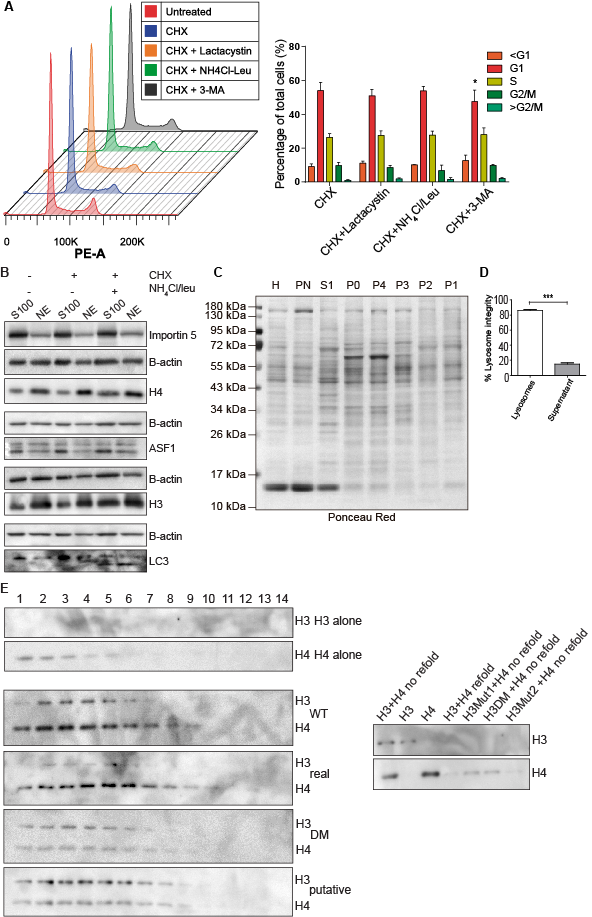


**Supplementary Figure 1. A. Cell cycle analyses upon inhibition of different degradation pathways.** HeLa cells were incubated for 1 h with 100 μg/mL CHX plus nothing, 10 μM Lactacystin, 20 mM NH_4_Cl + 200 μM leupeptin, and 10 mM 3-MA, as indicated. Left, Cell cycle profile. Right, percentage of cells in each cell cycle stage. The standard deviation was obtained from three independent experiments. *p-value<0.05, Student´s t-test. **B.** Immunoblot of 5 µg of cytosolic and nuclear extracts derived from HeLa cells incubated for 1 h with 100 μg/mL CHX plus nothing or 20 mM NH_4_Cl + 200 μM leupeptin, as indicated. **C.** **Lysosome extraction from the rat liver.** Ponceau Red analyses loading 30 µg of the different fractions collected, H: liver homogenate, PN: post-nuclear fraction, S1: supernatant of PN, P0: input of the endoplasmic reticulum, mitochondria and lysosomal fractions, P1: lysosomes enrichment in the CMA activity (CMA+), P2: lysosomes mix of CMA+ lysosomes and those with low CMA activity (CMA-), P3: lysosomes and light mitochondria, and P4: mitochondria and endoplasmic reticulum. **D. Lysosomal latency.** The assay measured the lysosomal enzyme β-hexosaminidase activity in supernatants to control the integrity of the lysosomal CMA+ fractions. The graph shows mean + SD of the lysosomal latency as percentage from 15 independent experiments. ***p<0.001, Student´s t-test. **E. H3-H4 tetramer formation with histone H3 mutants on the KFERQ motif.** Ten µg of histone H3 wild-type or mutants on the KFERQ motif were mixed with 10 µg of histone H4 in unfolding buffer (7 M guanidinium HCl, 20 mM Tris-HCl pH 7.5, 10 mM DTT) and incubated for 1 h at 4℃. Samples were then dialyzed against refolding buffer (2 M NaCl, 10 mM Tris-HCl pH 7.5, 1 mM Na-EDTA, 5 mM 2-mercaptoethanol) and then centrifuged at 15,000 rpm for 20 min at 4℃. The supernatant containing H3-H4 tetramers was loaded onto a 5-30% glycerol gradient in a buffer containing 2M NaCl, 10 mM Tris-HCl, pH 7.5, 1 mM Na-EDTA, 5mM 2-mercaptoetanol, and ultracentrifuged for 16 h, 4℃, 26,000 rpm. Fractions of 500 µL were collected. Aliquots derived from the fractions were analyzed by western blot. Because we did not observe histone H3 when centrifuged alone, we loaded 10 µL of the last fraction that contained aggregates.


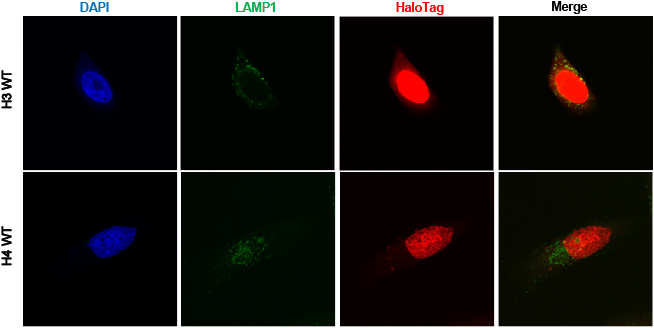


**Supplementary Figure 2.**  Representative confocal microscopy images of immunofluorescence in HeLa cells expressing HaloTag-H3 (HT-H3) or HaloTag-H4, stained with HaloTag-TMR ligand (red), DAPI (blue), immunostained against LAMP1 (green), and the merge of green and red channels.


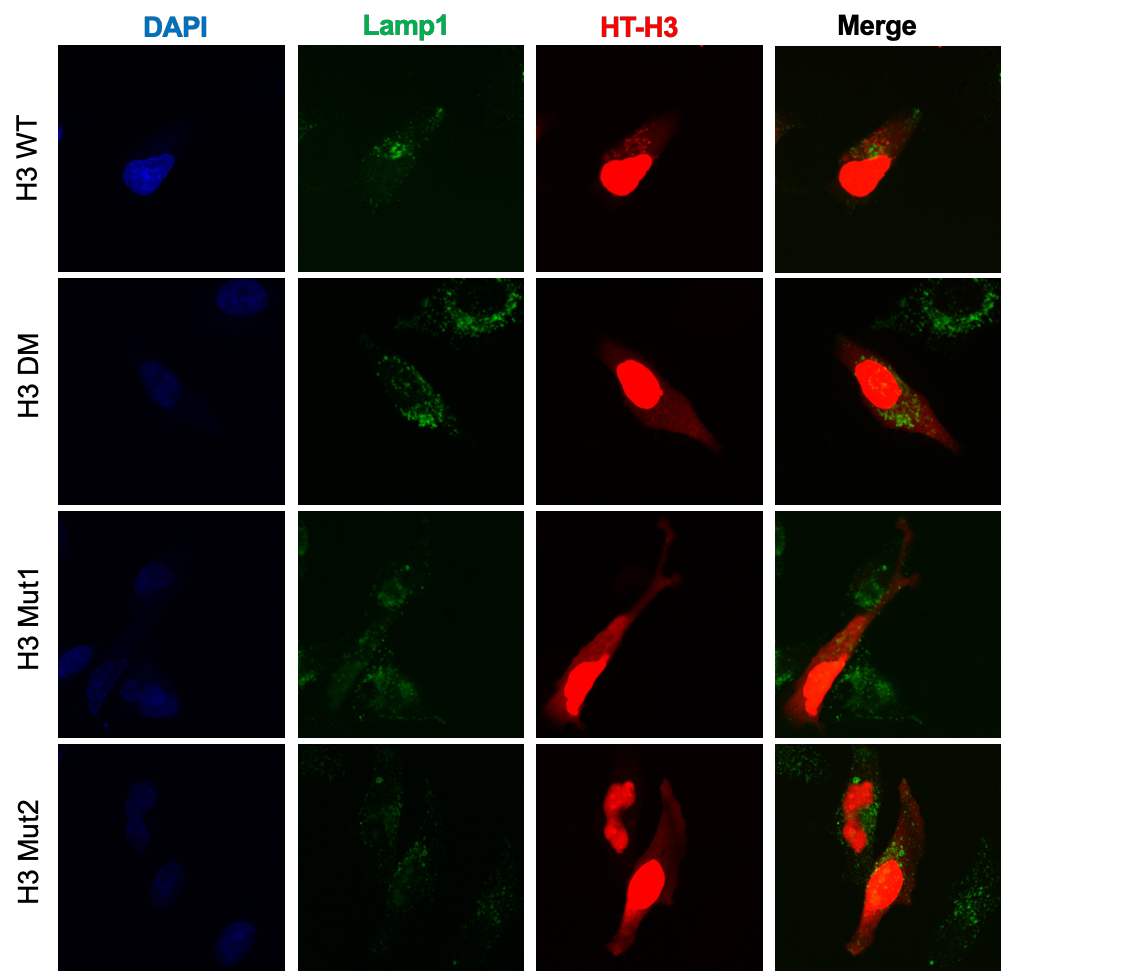


**Supplementary Figure 3.** Representative confocal microscopy images of immunofluorescence in HeLa cells expressing HaloTag-H3 (HT-H3) and mutant versions of HaloTag-H3 (H3DM; H3Mut1 and H3Mut2), stained with HaloTag-TMR ligand (red), DAPI (blue), immunostained against LAMP1 (green), and the merge of green and red channels**.**
